# Ultrasensitive terahertz metamaterial sensor based on spoof surface plasmon

**DOI:** 10.1038/s41598-017-01781-6

**Published:** 2017-05-18

**Authors:** Xu Chen, Wenhui Fan

**Affiliations:** 1 State Key Laboratory of Transient Optics and Photonics, Xi’an Institute of Optics and Precision Mechanics, Chinese Academy of Sciences, Xi’an, 710119 China; 2 University of Chinese Academy of Sciences, Beijing, 100049 China

## Abstract

A planar terahertz metamaterial sensor consisting of a corrugated metal stripe perforated by three rectangular grooves is proposed and investigated numerically. Due to the formation of Fabry-Perot resonance of the spoof surface plasmons mode on the corrugated metal stripe, the extremely sharp resonance in transmission spectrum associated with strong local field enhancement and high quality factor can be realized and exploited for ultrasensitive sensing. Since the intense interaction between electromagnetic waves and analyte materials, the frequency sensitivity of 1.966 THz per refractive index unit and the figure of merit of 19.86 can be achieved. Meanwhile, the film thickness sensitivity of this metamaterial sensor is higher than 52.5 GHz/μm when the analyte thickness is thinner than 4 μm. More interestingly, we find that the metal thickness has a great effect on the sensor performance. These findings open up opportunities for planar metamaterial structures to be developed into practical sensors in terahertz regime.

## Introduction

Metamaterials (MMs) are periodic arrangement of artificially structured materials, in which the dimension of the unit cell is much smaller than the incident wavelength, have attracted much attention in the past decades^1, 2^. By tailoring geometry of the unit cell, MMs can create effective mediums with controllable permittivity and permeability. Many exotic properties, such as negative refraction^1^, perfect lensing^3^, cloaking^4^, could be realized, which have tremendous potential applications in super lens^5^, near perfect absorbers^6, 7^, modulators and filters^8, 9^. Terahertz (THz) waves, with low photon energy and nonionizing properties, have widely applications in medical imaging, remote sensing, astronomical radiation detection, and biomedical analytics^10–12^. Importantly, many chemicals and molecules have collective vibrational and rotational modes in THz regime, resulting in unique absorption spectral features^13, 14^, which can be applied to fingerprint detection and identification.

Recently, MMs based THz devices by designing their structural dimensions have been used in chemical and biological sensing to micro-environmental changes^15, 16^. Due to the low quality (*Q*) factor resonance caused by radiation losses and the lack of strong electromagnetic interaction^17, 18^, the sensitivity and figure-of-merit (FOM) of these reported sensors are relatively low. With the strongly confined electromagnetic field and sharp spectral resonance features, MMs structures can strengthen field-matter interaction and improve *Q* factor, which is similar to surface plasmon polaritons (SPPs) sensing at visible wavelengths^19, 20^. However, the properties of SPPs sensing at visible wavelengths is not able to be transferred to THz frequencies directly, because the metal cannot bind THz waves tightly in metal surface. Luckily, highly confined electromagnetic surface waves supported by metal surface with corrugated structure at subwavelength scales has been proposed and demonstrated at THz frequencies^21, 22^. Similarly to the SPPs in visible regime, these surface modes are usually termed as spoof surface plasmons (SSPs)^21^. More recently, with large local field enhancements and sharp resonance features, the SSPs-based stripe antennas in THz band have also been studied^23, 24^. The tightly confined SSPs mode^25^ indicates that they could be very sensitive to surrounding dielectric environment, offering another important sensing technique in THz frequencies. Many THz MMs sensors have been proposed as metal-dielectric-metal configurations^26, 27^, planar structures based on breaking symmetry^28, 29^ and graphene based structures^30, 31^, but the planar THz MMs structures with the SSPs-based sensing are rarely investigated.

In this paper, for the first time to the best of our knowledge, we propose an ultrasensitive THz planar MMs sensor based on the excitation of the sharp SSPs resonance mode. The extremely sharp resonance with narrow linewidth and high *Q* factor can be utilized for refractive index and thickness sensing. Under certain conditions, the MMs sensor can realize much superior performance in the THz regime, such as sensitivity and FOM, which are higher than previously reported works based on planar MMs structures^28, 30, 32^. Furthermore, we find that the metal thickness has an important effect on the sensor performance.

## Structure design and principle

**Figure 1:**
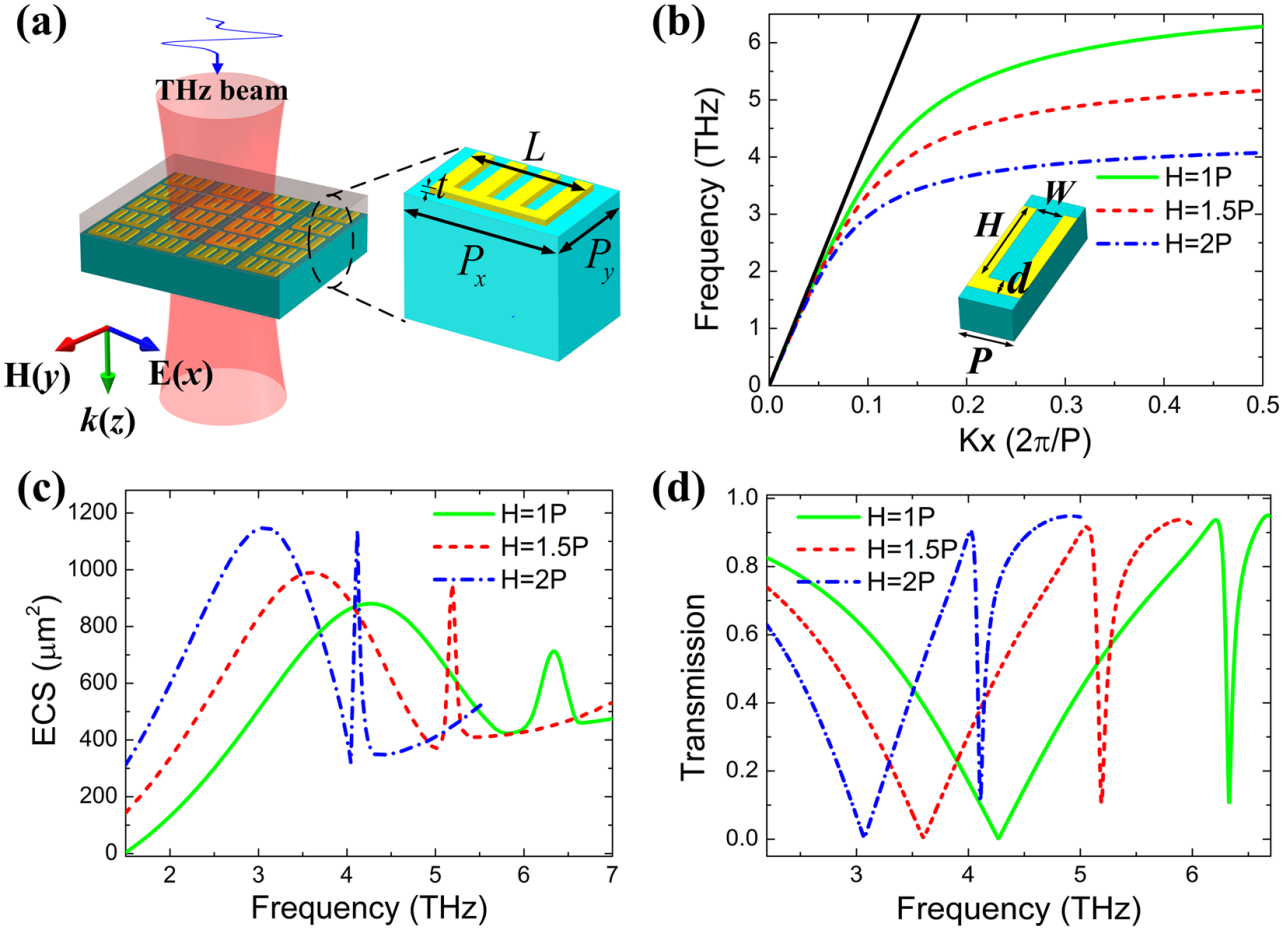
(**a**) Schematic diagram of the MMs structure covered by analyte layer, the zoom in is the unit cell with geometrical parameters: period *P*_*x*_ = 28 μm and *P*_*y*_ = 18 μm, metal thickness *t* = 2 μm, length *L* = 3.5*P*. (**b**) The calculated dispersion curves of the SSP modes for the period groove array with different groove depth *H*, groove width *W* = 0.5 *P*, *P* = 6 μm, distance *d* = 2 μm. The calculated ECS spectra (**c**) and transmission spectra (**d**) of the MMs structure with different groove depth *H*.

Figure 1(a) schematically illustrates the geometry of the MMs structure, in which the unit cell is composed of a corrugated metal stripe perforated by three rectangle grooves periodically arrayed in *x* and *y* direction. The substrate is polyimide (PI) with dielectric constant of 3 and loss tangent of 0.005^33^, which shows high transparency in the THz range^34^. The geometrical parameters of the unit cell are described in Fig. 1(a) and (b).

Based on previous reports, the infinite periodically groove array could support a well-confined SSP mode with a relatively low propagation loss^35^. So, the SSP frequency is conformed to the dispersion relation of SSP mode, whose asymptote frequency is mainly controlled by the groove depth *H* ^36^. In Fig. 1(b), based on eigen-frequency solver, we calculated dispersion relation of the SSP mode for this periodic groove array with different groove depth *H*. As a comparison, the black straight line is given from the light in the background medium which is assumed to be homogeneously PI. One can clearly observe that the grooved metal stripe exhibits a well-pronounced surface plasmon-like behavior. The wave vector of the SSP modes deviates from the black straight line with the frequency increasing, and the dispersion curves becomes flat when the frequency asymptotes to a certain value. With the groove depth *H* increasing from 1*P* to 2*P*, the asymptote frequency of dispersion curves decreases from 6.32 THz to 4.10 THz. More importantly, the dispersion curve moves to the right when the groove height increases, showing the tighter modal confinement tendency.

The extinction cross section (ECS) of the MMs structure, which is the sum of absorption cross section and scattering cross section, is also investigated. It is a far-field parameter to determine the scattering properties of an object, representing the strength ratio of incident EM wave coupling to the object^36, 37^. To evaluate the localized SSP resonances in the MMs structure, we calculate ECS with different groove depth using the finite-element numerical simulations, based on commercial software CST Microwave Studio. The ECS calculation is performed using a normal incident plane wave excitation, and a broadband far-field monitor is selected to record the field information. So we obtain the frequency-dependent ECS spectrum. As shown in Fig. 1(c), two resonance peaks in the ECS spectra for different groove depth can be observed, the first one has a broader resonance, while the second is much sharper. With *H* increasing, both resonances not only shift to the lower frequency, but also become narrower, which can be attributed to the tighter modal confinement for larger groove depth. The transmission spectra of this MMs structure were simulated with different groove depth *H* as shown in Fig. 1(d). Two transmission dips can be observed, the first one is a broad resonance; the second one is a sharper resonance and has an asymmetric Fano resonance line shape. As the groove depth *H* increasing, the frequencies of both dips are red shift and the corresponding bandwidth become narrower, leading to *Q* factor increasing. Therefore, from the qualitative point, the sensing mechanism of the MMs structure is due to the formation of the Fabry-Perot (FP) resonance modes which arisen from the excitation of the SSP mode propagating back and forth along the corrugated metal stripe and reflecting at both ends, these resonance modes with enhanced light-matter interaction and high *Q* factor can be used for sensing, which is similar to those of nanostripe antennas working at optical frequencies^24, 38^.

## Results and Discussion

**Figure 2:**
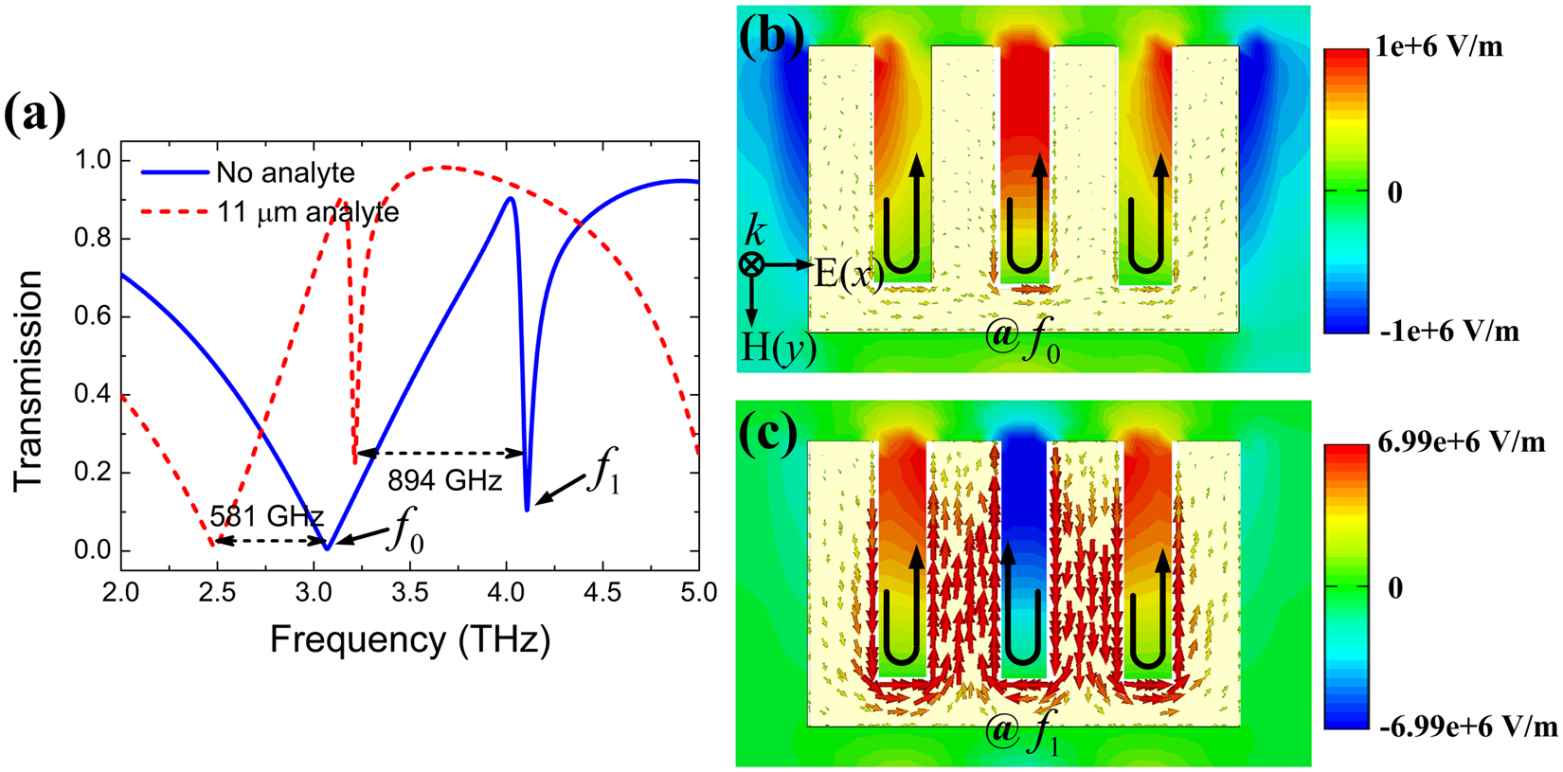
(**a**) Transmission spectra of the MMs structure without analyte (blue solid line) and with 11 μm thick analyte layer (red dotted line), the refractive index of analyte is *n* = 1.6. (**b**,**c**) Electric field and surface current distributions of the MMs structure at frequencies *f*_0_ and *f*_1_ without analyte layer. The black arrows indicate the surface current direction.

First of all, the transmission spectra of the MMs structure have been simulated and presented in Fig. 2(a), with the metal thickness of 2 μm, and the groove depth *H* = 2*P* was chosen because of the sharpest resonance performance. For the bare MMs structure without analyte layer (blue solid line), two resonant dips can be observed, and the sharp resonance features a small bandwidth 71.35 GHz at 4.10 THz, thus the *Q* factor is 57.46. With an analyte layer thickness of 11 μm and refractive index of 1.6 coating on the structure, there are clear red shift in the resonance frequencies of 581 GHz and 894 GHz for the broad and the sharp resonances, respectively. The red shift of the resonance takes place as a result of changing the effective refractive index of the environment^28^, and can be utilized to identify the type of analyte materials. To interpret the resonance mechanism, we simulated the electric field (*E*_*x*_) and surface current distributions at frequencies *f*_0_ and *f*_1_ for the bare MMs structure, as shown in Fig. 2(b) and 2(c). For the broad resonance *f*_0_, it has a mode profile quite like a regular half-wavelength dipole mode, resulting from a small deviation of the SSP wave vector to that of the light line shown in Fig. 1(b) ^23^. For the sharp resonance *f*_1_, it is obviously a higher order (or third-order) mode resonance. More THz electric fields are confined within the grooves at this sharp resonance frequency than that of the broad one, indicating the stronger interaction between THz waves and the analyte at the sharp frequency. As for the surface current distributions, the broad resonance *f*_0_ has the uniform current direction for all grooves and the current density is relatively weak; while the sharp resonance *f*_1_ has anti-parallel current distributions in adjacent grooves which ensure extremely low radiation losses of the incident wave and the current density is larger. This also demonstrates that the broad and sharp resonances are respective the dipole and high order mode resonance, respectively.

**Figure 3:**
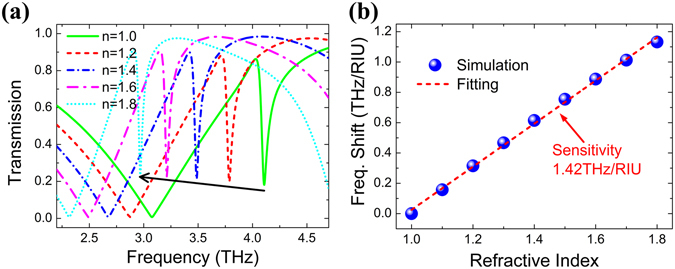
(**a**) Transmission spectra with refractive indices of the analyte changing from 1.0 to 1.8. (**b**) Frequency shift versus different refractive indices of the analyte, blue spheres are the simulation data, and red dotted line shows the linear fitting to the simulation data.

The dependence of the transmission spectra on the refractive indices of analyte was calculated and shown in Fig. 3(a), with its thickness fixed as 11 μm. Both resonance frequencies of the broad and the sharp are gradually red shifting with refractive index increasing from 1.0 to 1.8 in steps of 0.2, arising from the dynamic-capacitance changes with the analyte layer. The range of refractive indices represents a series of important materials for THz sensing application and it also covers biomolecules varied from 1.4 to 1.6 in DNA and 1.6 to 2.0 in RNA^30, 32^. From Fig. 3(a), the total shifts of the broad and the sharp resonance frequencies are 0.762 THz and 1.139 THz, with the refractive index from 1.0 to 1.8. So, owing to the more electromagnetic confined capacity discussed aforementioned and the larger resonance shift in response to the analyte refractive index, the sharp resonance dip is more suitable for sensing, which is used as the sensing frequency in the following calculations. In Fig. 3(b), we also calculated the frequency shift (*FS*) with different refractive indices of the analyte, where *FS* = *f*_*n*_ − *f*_*n*0_, *f*_*n*_ is the sharp resonance frequency with the analyte refractive index *n* and *f*_*n*0_ is the reference frequency with refractive index *n*_0_ = 1. It can be seen that the frequency shift increases linearly with the refractive index increasing. Since the frequency sensitivity (*S*) is defined as the slope of the linear fitting function of the frequency shift, the frequency sensitivity can be calculated by fitting the simulation data. Note that the fitting function is *FS* = −1.395 + 1.42*n*, thus the frequency sensitivity 1.42 THz per refractive index unit (RIU) can be obtained.

**Figure 4:**
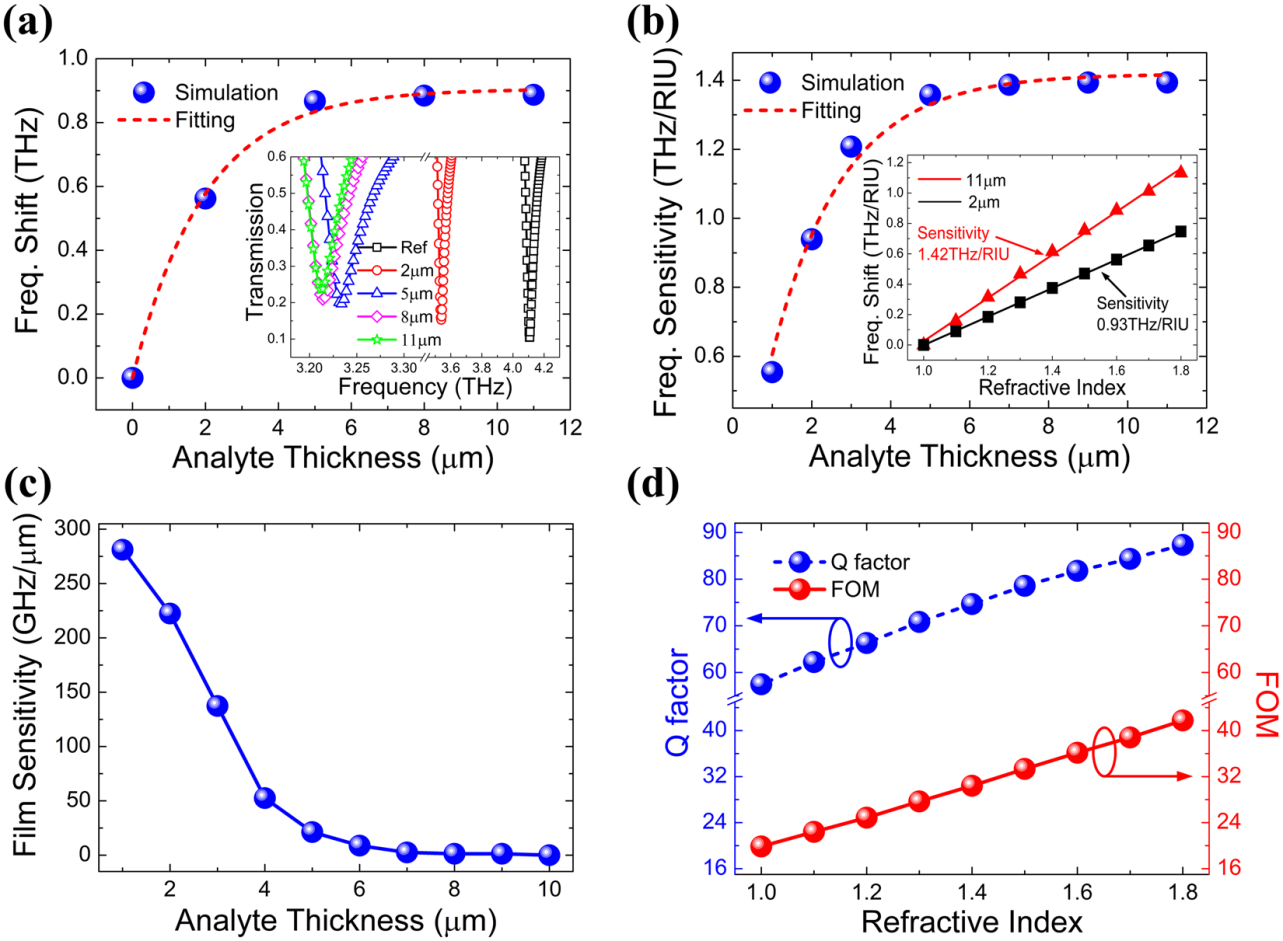
(**a**) Frequency shift of the MMs structure with different analyte thicknesses, the red dotted line is the exponential fitting to the simulation data (blue sphere). The inset shows the simulated transmission dips with different analyte thicknesses. (**b**) The frequency sensitivity versus different analyte thicknesses, the inset shows the sensitivity with 2 μm and 11 μm analyte layer. (**c**) The film thickness sensitivity versus analyte thicknesses. (**d**) Variation of *Q* factor and FOM with different refractive indices of the analyte.

Next, the effect of the analyte thickness on the frequency sensitivity of the sensor has been studied comprehensively, with analyte refractive index of 1.6. In Fig. 4(a), we plotted the frequency shift (*FS*) versus different thicknesses of the analyte layer, as *FS* = *f*_*t*_ − *f*_*Ref*_, where *f*_*t*_ is the resonance frequency when the analyte thickness is *t* and *f*_*Ref*_ is the resonance frequency without analyte layer. In the inset of Fig. 4(a), one can see clearly that, as the analyte thickness increases, the resonant frequency dip initially red shifts quickly and then stop moving at a largest thickness of 11 μm, indicating that increasing thickness no longer affects the resonance frequencies. Meanwhile, the simulation data was fitted by exponential function and it matched reasonably well. The fitting function was described by *FS* = 0.905–0.905*e* ^−0.509*t*^, and the total frequency shift saturated at about 894 GHz. Moreover, the exponential fitting function indicates that the fringing fields decay exponentially from the top surface of the MMs structure^27^. Similarly, the frequency sensitivity dependence on the analyte thicknesses was also calculated. As shown in the inset of Fig. 4(b), we first investigated the dependence of the resonance frequency shift on the refractive index variation for the sensor with two different analyte thicknesses of *t* = 2 μm and *t* = 11 μm. A significant increase of the sensitivity can be achieved when the analyte thickness increases from *t* = 2 μm to *t* = 11 μm. Furthermore, the relationship of the sensitivity with the different analyte thickness was clearly plotted in Fig. 4(b). The frequency sensitivity increases exponentially with the analyte thickness increasing and eventually saturates at about 11 μm, highlighting the electric field extended up to 11 μm in the analyte layer on top of the MMs structure. The increase of the frequency sensitivities can also be represented by the exponential fitting curves, expressed as *S* = 1.425–1.425*e* ^−0.556*t*^, and the largest sensitivities is 1.42 THz/RIU.

On the other hand, for the urtrathin analyte layer, we find that this MMs structure has a good performance on the film thickness sensing. The film thickness sensitivity *S*(*t*) of the MMs sensor can be calculated by ref. 39

1 $$S(t)=\frac{{f}_{t-1}-{f}_{t+1}}{{\rm{\Delta }}t}(\text{GHz}/\mu m)$$

where *f*_*t*−1_ and *f*_*t*+1_ are the resonant frequencies at the film thickness of *t* − 1 and *t* + 1, Δ*t* = 2 μm. Figure 4(c), indicates that the film thickness sensitivity is dependent on the analyte thickness. When the film thickness is 4 μm, the film sensitivity can be up to 52.5 GHz/μm, and it approximately linearly increases with the film thickness thinner than 4 μm. Thus, the ultrathin film thickness sensing can be realized. For terahertz-time domain spectroscopy, if the frequency domain resolution is *D*_*f*_, then the film thickness resolution *D*_*t*_ of the MMs sensor can be calculated as *D*_*t*_ = *D*_*f*_/*S*(*t*), indicating that even a very small changes of the film thickness can be also detected since the sensitivity is highly increased with the film thickness decreasing. Furthermore, to quantitatively describe the sensing performance, we calculated the FOM, defined as the ratio of sensitivity to full width at half maxima (FWHM) of the transmission spectrum, and the FWHM is inversely proportional to the *Q* factor^30^. Figure 4(d) plotted the variation of *Q* factor and the corresponding FOM as a function of the analyte refractive index, with the analyte thickness fixed as 11 μm. When the refractive indices increase from 1.0 to 1.8, the *Q* factors increase linearly from 57.46 to 87.32, which are really high *Q* values, indicating the enhanced detection accuracy and capability. The FOM also increase linearly from 19.86 to 41.76 with the refractive indices increasing, which is consistent with the relationship that the FOM is proportional to *Q* factor.

**Figure 5:**
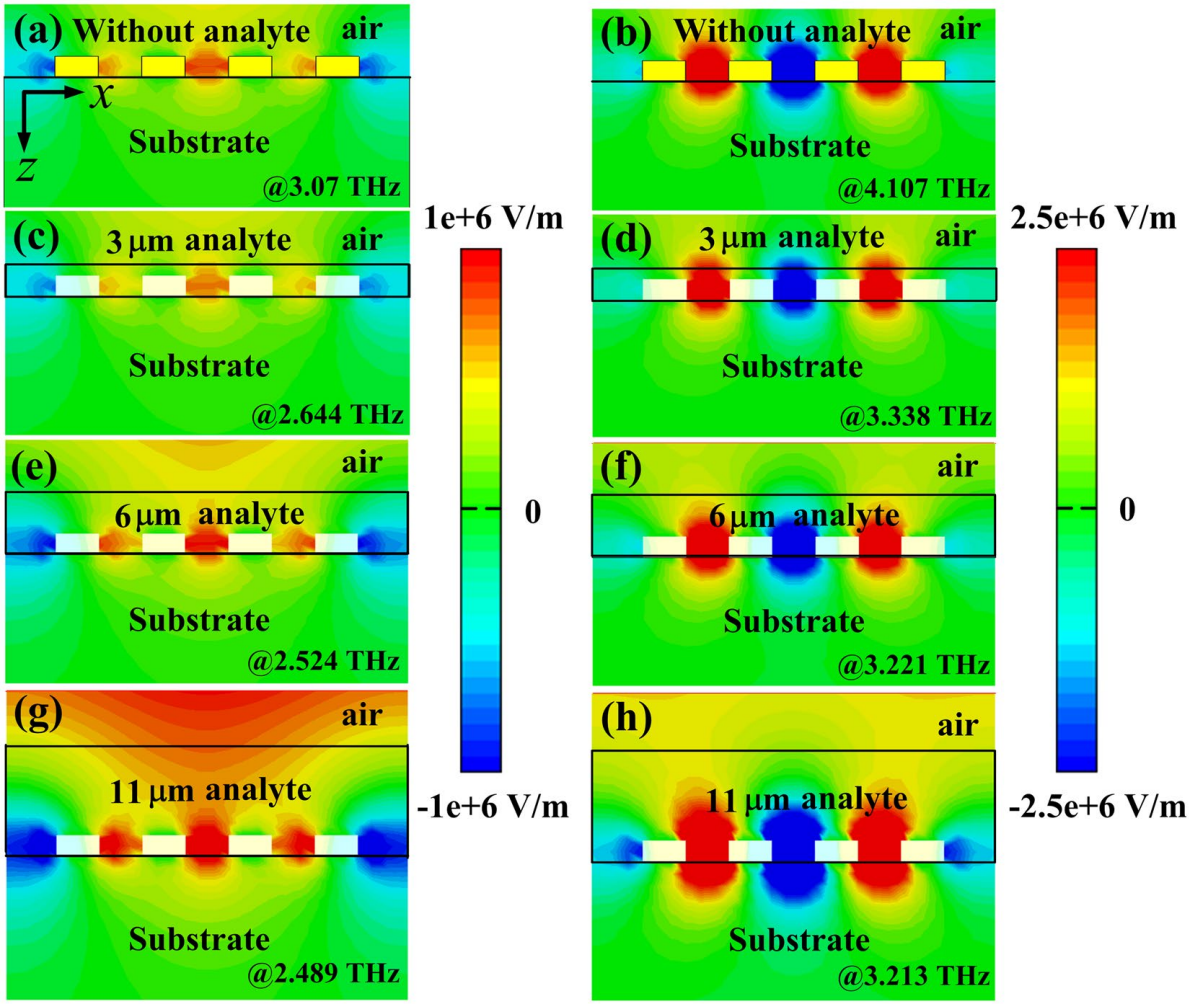
Electric field (*E*_*x*_) distributions at *x*-*z* plane with different analyte thicknesses. (**a**,**b**) 0 μm, (**c**,**d**) 3 μm, (**e**,**f**) 6 μm, (**g**,**h**) 11 μm, respectively. The left column is the broad resonance, and the right column is the sharp resonance.

To clearly explain the sensing mechanism versus the analyte thickness of this MMs sensor, we simulated the electric field (*E*_*x*_) distributions of the broad resonance mode and the sharp resonance mode under different analyte thicknesses (e.g., 0 μm, 3 μm, 6 μm, 11 μm), with the analyte refractive index fixed as 1.6. The left column of Fig. 5 is the electric field distribution of the broad resonance mode, and the right column is the sharp resonance mode. In the absence of analyte layer, it is clearly seen that the enhanced electromagnetic wave intensity is localized in the grooves and interface between the device and air. With the analyte layer coating on the structure, the enhanced resonant electric field is clearly squeezed to a smaller spatial extent along *z* direction, thus more fields are confined in the grooves. At the same time, the introduction of the analyte layer will change the resonance frequencies. As the analyte thickness increasing, more and more resonant field distributed in the analyte layer, whose refractive index is larger than air, leading to red shifting of the resonance frequencies. For 11 μm thickness analyte layer, the resonance electric field was completely localized in the analyte layer, as shown in Fig. 5(g) and (h), the resonant frequencies stop moving, and increasing analyte thickness has no influence on the resonance frequency, which agrees with the results shown in Fig. 4(a). Therefore, the enhanced electric field in the grooves along with the fringing field above the sensor surface plays a significant role in analyte sensing and explains the highly sensitive nature of the MMs structure. In addition, more electromagnetic waves are distributed at the sharp resonance than that of the broad one, indicating larger field enhancement and stronger field-matter interaction for the sharp resonance. This once again indicates the sharp resonance has better sensing performance than that of the broad one.

**Figure 6:**
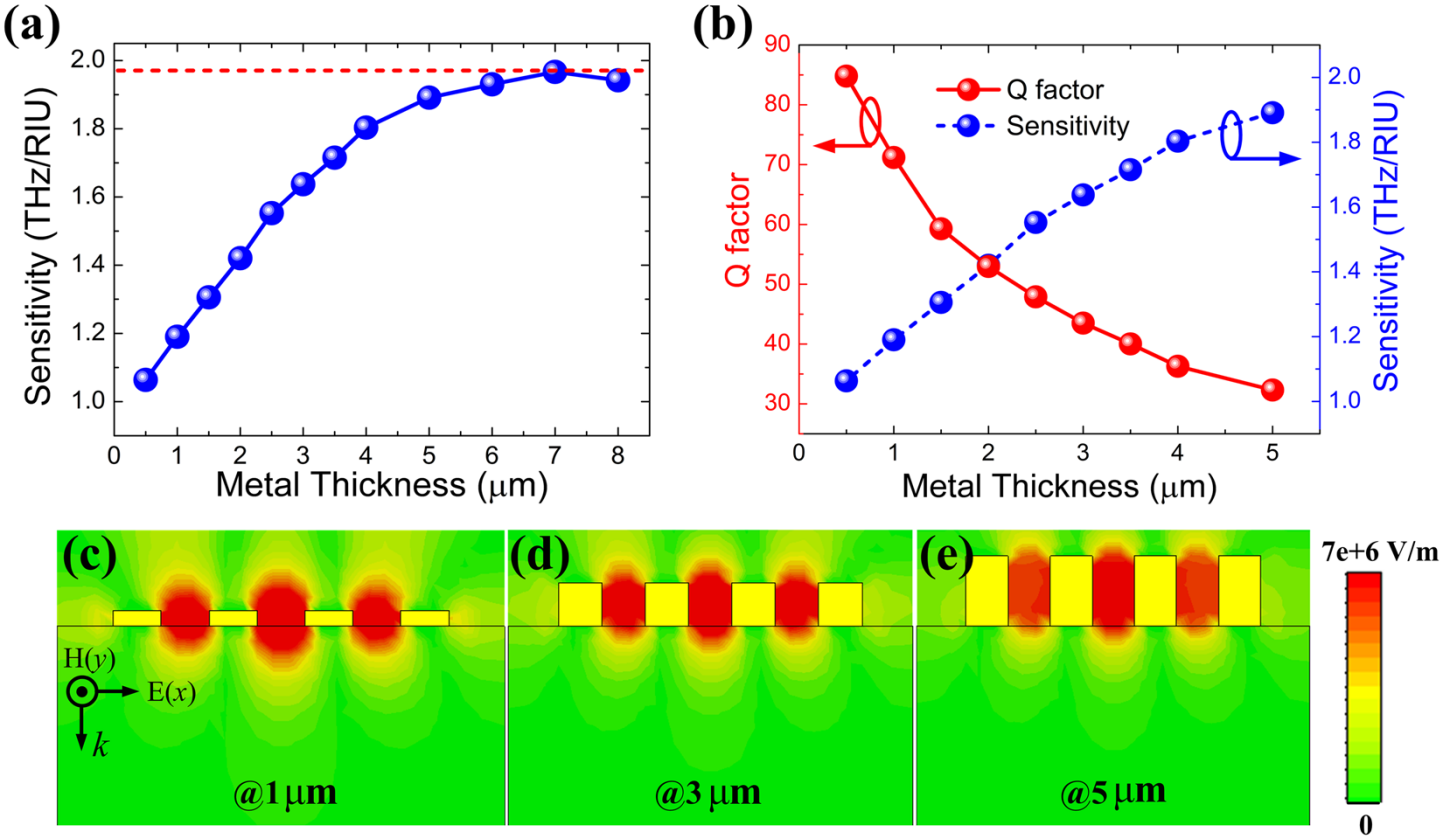
(**a**) Frequency sensitivity with varying the metal thickness of the MMs structure, the red dotted line is the maximum sensitivity. (**b**) The *Q* factor and sensitivity varied with the metal thickness. (**c**,**d**) Electric field intensity (|*E*_*x*_|) distributions of the sharp resonance mode with the metal thicknesses of 1 μm, 3 μm and 5 μm, respectively.

Finally, we find the metal thickness has an important effect on the frequency sensitivity of the MMs sensor, which has not been investigated by previous works yet. Figure 6(a) shows the changes of the sensitivity as a function of the metal thickness. With the metal thickness increasing, the sensitivity increases quickly at first and achieves the maximum value 1.966 THz/RIU when the metal thickness is 7 μm, and then decreases. The sensitivity value of 1.966 THz/RIU is higher than recently reported works based on THz MMs sensors^28, 32^. The *Q* factor with varying metal thickness has also been calculated in Fig. 6(b). Due to the loss of the metal, the *Q* factor decreases monotonously with the metal thickness increasing, indicating decreases of the FOM. To get a better sensing performance, as shown in Fig. 6(b), the metal thickness of the MMs sensor should be 2 μm and the corresponding FOM will be as high as 19.86, which is higher than Zhang *et al*. reported work^30^. The electric field intensity (|*E*_*x*_|) distributions of the sharp resonance modes have also been simulated to interpret the relationship of sensitivity and metal thickness, with the metal thickness of 1 μm, 3 μm and 5 μm for simplicity. From Fig. 6(c–e), it is clearly observed that the electric field intensity is more confined and localized in the metal grooves with the metal thickness increasing from 1 μm to 5 μm. And also, the stronger electromagnetic interaction between the THz waves and the analyte can be achieved with the enhanced electric field intensity, resulting in the sensitivity increase.

## Conclusion

In summary, we have theoretically demonstrated an ultrasensitive metamaterials sensor, in which the unit cell is composed of a corrugated metallic stripe perforated by three rectangle grooves. Due to the excitation of spoof surface plasmon modes in THz regime, the extremely sharp (or high-order) resonance can be generated and utilized for analyte sensing. More specifically, we deeply and comprehensively investigated the performance of this metamaterials sensor with different analyte thicknesses and refractive indices. Numerical results show that the high *Q* (57.46) factor and FOM (19.86) can be realized simultaneously, and the frequency sensitivity can be achieved as high as 1.966 THz/RIU when the metal thickness is 7 μm. Moreover, this device has highly sensitive sensing at very thin analyte thickness (i.e., the film thickness sensitivity is higher than 52.5 GHz/μm when the analyte thickness is thinner than 4 μm). Because of the compact planar metamaterials configuration, such designs can be realized and it will open up a new window for highly efficient THz biomedical sensors.

## Methods

### Numerical simulation

We model and simulate the unit cell structure by the commercial software CST Microwave studio 2012. Periodic boundary conditions are applied along the *x* and *y* directions, and perfectly matched layers are employed in the *z* direction. A plane wave with the electric field along the *x* direction normally incidents on the structure. We use frequency-domain solver to obtain the transmission spectra of the structure. A broadband far-field monitor is applied to record absorption cross section and scattering cross section to calculate the ECS spectrum. Based on eigenmode solver of the CST, the dispersion relation of the unite cell is calculated and analyzed. The electric field and surface current distributions are obtained based on the field monitor at resonance frequencies. The dielectric substrate is polyimide with dielectric constant of 3 and loss tangent of 0.005. The material of the metal is gold with an electric conductivity 4.6 × 10^7^ s/m.
